# Evaluation of Three Formulations of Culture Media for Isolation of *Brucella* spp. regarding Their Ability to Inhibit the Growth of Contaminating Organisms

**DOI:** 10.1155/2014/702072

**Published:** 2014-04-10

**Authors:** Acácia F. Vicente, João M. A. P. Antunes, Gustavo H. B. Lara, Mateus S. R. Mioni, Susan D. Allendorf, Marina G. Peres, Camila M. Appolinário, Fernando J. P. Listoni, Marcio G. Ribeiro, Jane Megid

**Affiliations:** Departamento de Higiene Veterinária e Saúde Pública (DHVSP), Faculdade de Medicina Veterinária e Zootecnia (FMVZ), Universidade Estadual Paulista (UNESP), 18618-970 Botucatu, SP, Brazil

## Abstract

Three culture media (*Brucella* agar, Farrell medium, and CITA) were compared for their effectiveness in inhibiting contamination and for isolating *Brucella* spp. One hundred lymph nodes from pigs (*n* = 50) and wild boars (*n* = 50) with lymphadenitis were collected in slaughterhouses in the State of São Paulo and were assessed on these three selective media for *Brucella* spp. All of the samples were negative for *Brucella* spp. on the three culture media. On the agar medium, fungal (70 plates) and Gram-positive bacterial (59 plates) contaminants were observed; in the CITA medium, the absence of fungal and Gram-positive bacteria on 15 plates was observed; no bacterial or fungal growth was observed on the Farrell media. The results demonstrated that the CITA and Farrell media inhibited the growth of contaminants better than the *Brucella* agar.

## 1. Introduction

*Brucella* is a genus of Gram-negative, facultative intracellular bacteria that do not multiply in the environment and are usually transmitted directly between hosts. These microorganisms are responsible for causing diseases with variable clinical signs, depending on the host and the* Brucella* species. Most* Brucella* spp. are zoonotic and widely distributed in the world [1]. According to the International Committee on Taxonomy Bacteria [2], the genus has six classic species:* B. abortus*,* B. melitensis*,* B. suis*,* B. canis*,* B. ovis,* and* B. neotomae*. This classification is primarily based on the pathogenic differences, preference for hosts, and phenotypic characteristics. Recently, the marine species* B. ceti*,* B. pinnipedialis, B. microti,* and* B. inopinata* were included in the genus.

*B. suis* has five biovars, of which 1, 2, and 3 mainly affect swine [3]. After infecting an animal, the bacteria pass through the respiratory, digestive, or genital mucosa, undergo phagocytosis, and reach local lymph nodes [1]. In swine, bacteremia occurs intermittently for several weeks. The bacteria settle mainly in the mandibular, gastrohepatic, internal iliac, and retropharyngeal ganglia as well as in the liver, kidneys, joints, and reproductive organs [4]. Among the clinical signs are abortion and infertility in females and orchitis in males. Occasionally, arthritis, laminitis, and abscesses of different sizes in organs and tissues may occur [5].

Lymphadenitis is one of the main diseases of swine in Brazil and causes significant economic loss due to the condemnation of affected organs and carcasses [6].

*B. abortus* and* B. melitensis *may infect swine, although* B. suis* is the most common pathogenic species in swine [7].

In affected swine,* Brucella* spp. may be isolated from vaginal discharge, placenta, fetus, and semen. After a necropsy,* Brucella* may be isolated from lymph nodes, liver, spleen, mammary glands, epididymis, and prostate [8].

The “gold standard” for the diagnosis of brucellosis is bacterial isolation. The OIE (Office International des Epizooties) [7] recommends the simultaneous use of selective media, including Farrell and modified Thayer-Martin media, for the primary isolation of* Brucella* species from animal samples. The Farrell selective medium inhibits the growth of most contaminants and is probably the most selective medium used for bacteriological diagnosis in laboratories worldwide. However, some antimicrobials present in this formulation inhibit the growth of some* Brucella* species. Modified Thayer-Martin medium shows greater sensitivity than Farrell medium; however, it does not inhibit contaminating microorganisms as well. For this reason, CITA medium was developed based on modified Thayer-Martin medium with different concentrations of antimicrobials and with the addition of Amphotericin B to inhibit contaminants without inhibiting the growth of* Brucella* spp. [8].* Brucella *agar medium is widely used in laboratories to diagnose brucellosis in Brazil and was used for the isolation of* B. suis *during an outbreak [9].

This study aimed to evaluate three selective culture media for* Brucella* spp. (*Brucella* agar, Farrell media, and CITA) to investigate the presence of* Brucella* spp. in the lymph nodes of pigs and wild boar, from slaughterhouses of the São Paulo state, Brazil, with lymphadenitis.

## 2. Materials and Methods

*Brucella *agar medium was prepared using* Brucella *medium base (OXOID) supplemented with 5% fetal bovine serum (Invitrogen). The same* Brucella *medium base was also supplemented with Farrell antimicrobials (OXOID) and 5% fetal bovine serum (Farrell medium). CITA medium was prepared using Blood Agar Base No. 2 (OXOID) supplemented with the following antimicrobials: vancomycin, colistin, nystatin, nitrofurantoin, and Amphotericin B (Sigma) and 5% fetal bovine serum. The proportions of the antimicrobials used were followed according to [8].

Lymph nodes from* Brucella*-negative pigs were macerated in a 1 : 10 dilution with buffered saline solution and subsequently used as negative controls. As a positive microbiological control to evaluate the culture media, these negative lymph nodes were macerated and contaminated with* B. canis, B. ovis, B. abortus, B. abortus *B19, and* B. abortus *Rb51. For the contaminations,* Brucella canis*,* Brucella ovis,* and* Brucella abortus *species were diluted to scale 1 of the McFarland standards, and the initial concentrations of* B. abortus* B19 (Biovet) and* B. abortus* Rb51 (Intervet-Shering-Plough Animal Health) were the recommended dilutions for the vaccines used. The initial bacterial suspensions were diluted 1 : 10 with TE buffer and used for lymph node contamination. A total of 180 *μ*L of the macerated lymph node with 20 *μ*L of each bacterial suspension was uniformly distributed on each plate using a Drigalski spatula.

In addition, 100 lymph nodes, 50 from pigs and 50 from wild boar with lymphadenitis, were collected in slaughterhouses in the state of São Paulo, Brazil. In the laboratory, these lymph nodes were macerated in a 1 : 10 dilution with sterile saline solution for microbiological analysis.

For this analysis, three different culture media were used:* Brucella *agar, Farrell medium, and CITA medium. For the controls and for the samples, two plates of each medium were used; one plate was maintained at 37°C in a normal atmosphere and another plate was maintained at 37°C with 10% CO_2_ and high humidity. The plates were observed daily and considered negative if there was no bacterial growth by the 14th day. Colonies that grew on the plates were evaluated by morphology, growth period, and Gram staining [10].

## 3. Results and Discussion

The growth of* B. canis*,* B. ovis*,* B. abortus*,* B. abortus* B19, and* B. abortus* Rb51 was observed in the 10 plates of* Brucella *agar medium plated with lymph nodes contaminated with the different* Brucella *spp. (controls). In 6 of these plates, fungal growth was observed, demonstrating a lack of inhibition of fungal contamination. The results of the analyzed lymph nodes (100 lymph nodes corresponding to 200 plates) showed 70 plates with fungal growth on* Brucella *agar medium, 39 from pigs and 31 from wild boar. The growth of these microorganisms is due to the absence of the antimycotic cycloheximide, which is present in the Farrell supplement and inhibits the translation of mRNA by ribosomes, preventing fungal protein synthesis [8]. Fungal growth may also inhibit bacterial growth, leading to a reduction in the sensitivity of the bacteriological diagnosis [11]. Therefore, selective culture medium is recommended for the isolation of* Brucella* spp. from contaminated materials. On the same medium, there was bacterial growth from 29 swine lymph nodes (6 with CO_2_, 6 without CO_2_ and 17 in both conditions of culture) and in 30 plates of wild boar samples (5 with CO_2_, 7 without CO_2_, and 18 in both conditions of culture). For these cultured bacteria, Gram staining and biochemical differentiation tests were performed. All of the samples contained Gram-positive bacteria, including* Mycobacterium* spp.,* Rhodococcus equi,* and* Streptococcus* spp. [12].

The controls of* Brucella* spp. also grew properly in CITA medium. However, in this medium no fungal growth was observed. The same result was also observed in the field samples. This result is due to the addition of the antimicrobial amphotericin B, which interacts with a steroid present in the membrane of the fungus and causes the loss of selective permeability of the membrane and cytoplasmic components [8]. On 11 plates of pig lymph nodes (2 with CO_2_, 3 without CO_2_, and 6 in both conditions of culture) and on 4 plates of wild boar lymph nodes (3 with CO_2_ and 1 without CO_2_), Gram-positive bacteria were observed on this medium because the antimicrobials present did not inhibit bacterial growth, with the exception of* Mycoplasma* species [8].

In the control material contaminated with* Brucella* spp., only the growth of* B. ovis* was inhibited on Farrell medium. The growth of the other species of* Brucella* and the absence of contaminants on the plates were verified. There was no bacterial or fungal growth on any of the plates cultured with material from the lymph nodes of pigs or wild boar. Farrell medium prevents the growth of fungal and commensal bacteria that commonly contaminate samples during collection and the Gram-positive bacteria that grow on* Brucella *agar and CITA medium. This is the best medium for inhibiting the growth of contaminants. However, some antimicrobials present in its formulation also inhibit the growth of* B. ovis* and make it difficult to grow* B. melitensis*,* B. suis*, and some strains of* B. abortus* [13]. In the case of* B. melitensis*,* B. abortus,* and* B. suis,* the inhibition is most likely due to high concentrations of nalidixic acid and bacitracin [11, 13].

Although* Brucella* strains were not isolated from the lymph nodes of pigs and wild boars evaluated here, there are many reports of pigs being seropositive for brucellosis in Brazil [14–16] and reports of brucellosis outbreaks [9]. The presence of positive animals is a concern; therefore, it is extremely important to use appropriate culture media for* Brucella* spp. to allow accurate detection at different stages of the production chain, from the raising and slaughtering of animals, where there is a risk of infection of humans through contact with animals and carcasses, to the sale and use of meat, which poses a risk for humans from inadequate food preparation.

## 4. Conclusion

The combined use of CITA and Farrell media showed good results, inhibiting contaminants in pig and wild boar lymph nodes collected from slaughterhouses and enabling the isolation of* Brucella* species from lymph nodes that were experimentally contaminated. Our results support the associated use of these media for* Brucella* spp. isolation.
